# The Treatment of Chancroid and Syphilis

**Published:** 1884-08

**Authors:** John Ashhurst


### Selections.

### THE TREATMENT OF CHANCROID AND SYPHILIS.

If any constitutional treatment is demanded in chancroid, it is such as is indicated by the general condition of the patient. Chancroid requires local treatment, but as syphilis is a constitutional affection, its treatment is constitutional or general. Local treatment is required for certain manifestations of syphilis, but the treatment, *par excellence*, is constitutional.

Speaking first of the treatment of chancroid, we may recognize three plans which have been adopted:

First, that form of treatment which aims to abolish the whole thing at once, that is, by excision. There are certain maladies in which, by this plan, we can get rid of the disease entirely, as in the case of certain tumors. So a local disease, which has begun in one or more spots, should theoretically be removable by cutting away the diseased tissue. This plan has, however, been tried and found wanting. The great objection to it is that the wound almost inevitably becomes inoculated by the chancroidal matter, and that the resulting sore is larger than the first one was, thus rendering the ultimate condition of the patient worse instead of better.

The second form of treatment, and that which I advocate, is one which aims not to remove the disease at once, but to favorably modify its future progress. This is the treatment by cauterization. By destroying the surface of the chancroidal ulcer we remove its virulent qualities and leave a healthy granulating sore. The caustic application removes the tendency to spread, and converts the ulcer into a healthy granulating surface. In speaking of this tendency to spread, I refer to one of the most prominent features of chancroid, its auto-inoculability, in which it differs from the initial lesion of syphilis. Chancroid is auto-inoculable indefinitely, and I believe that cauterization very much diminishes, if it does not destroy, this property, although the pus from the chancroid is still contagious. It seems to lose, after cauterization, to a great extent, that quality which causes it to spread to other parts on the same person. In the choice of a caustic, my preference is for fuming nitric acid, applied by means of a piece of soft wood, such as the end of a match-stick. Another plan is to apply the acid by means of a glass brush, but I do not think this as desirable. Every cranny should be cauterized. Any part that escapes retains its quality of furnishing auto-inoculable pus, and the whole surface may return to its former condition; therefore, cauterization must be thorough if it is practiced at all. When the slough, produced by the caustic, separates, the surface soon granulates and heals, but the pus is contagious to the last. If the fear of pain deter the patient from submitting to cauterization, general anæsthesia may be properly employed, or the surgeon may first make an application of carbolic acid, which produces local anæsthesia, and apply the nitric acid afterwards. It may be necessary to repeat the operation.

There are other modes of effecting cauterization. One is the use of the carbo-sulphuric paste, recommended by French surgeons. This forms a crust, which I think is a disadvantage, as concealing the parts beneath. The solution of acid nitrate of mercury may be used, but if applied over an extensive surface it may cause salivation. It is not as well adapted to the purpose as nitric acid. The actual cautery also has strong advocates; it may be employed either with the simple hot iron, or with the Paquelin's or the galvanic cautery. These modes of cauterization are effective in cases of serpiginous chancroid—in the latter I think the hot iron the best application that can be made. The material used by many practitioners a few years ago, the nitrate of silver, is inefficient, and, in my judgment, has nothing to recommend it. Then for the after-dressing, after cauterization has been employed, we can use plain water, or lime water, or black-wash, or a solution of salicylic acid, or what is known as the "nitric acid wash" (nitric acid *f* ʒj; water Oj), which is much used as a dressing in New York. When the chancroid is on a mucous surface, as in the female organs, or in any situation in which it is kept moist, a simple dry dressing of absorbent cotton or dry lint may be used; but where the chancroid is exposed, dry dressings are apt to become adherent, and wet applications are better. The dressing above all others which I think deserves attention is iodoform. It is a comparatively recent remedy in these cases, and I think that it is the best application that can be made after thorough cauterization has been effected. It can be used in various ways, by simply dusting the finely-powdered drug over the surface, or as a wet dressing in the form of an alcoholic solution with glycerine, viz.: Iodoform ʒss; alcohol *f* ʒij; glycerine *f* ʒvj. Or it may be used in the form of an ointment, xv to xxx grs. to the ounce, or as an etherial solution which evaporates, leaving a thin film of iodoform over the surface. An old remedy, which formerly had great reputation in these cases, was aromatic wine, but I do not think it is as efficient as iodoform. Another re...edy, which is quite a novel one, is resorcin, an article of the phenol series. Great advantage has been claimed for it. Pyrogallic acid has also been used, as has the subnitrate of bismuth and various other dry powders. In the female, dressings, of course, must be applied with the aid of the speculum.

In chancroids at the meatus, I commonly use a solution of nitrate of silver (xxx grs. to *f* ℥j), since the contraction after the use of nitric acid might be objectionable in this situation. At the frænum some special precautions may be required. Deep cauterization here may be followed by bleeding, and it has been proposed to prevent this by the previous application of ligatures, tying the frænum above and below the seat of disease, or by employing the actual cautery. For chancroids beneath the prepuce, when this can be retracted, the best plan is to cauterize the sores and dress them in the ordinary way, either replacing the prepuce afterwards, or allowing it to stay retracted, as may be thought most convenient. If, however, the prepuce cannot be retracted, then the surgeon may inject a strong solution of nitrate of silver, or, which I prefer when it can be done, may pack the space between the prepuce and glans penis with lint saturated with a solution of nitrate of silver (gr. xx to *f*℥j). Whenever it is necessary to circumcise the patient, of course the wound should be cauterized, as it will otherwise become inoculated and itself converted into a large chancroid. As for urethral chancroids, which are very rare, cauterization cannot be employed, as increasing the risk of stricture; absorbent cotton may be used as a dressing, taking care to have a thread attached by which the dressing may be withdrawn. About the rectum and anus, chancroids may be treated by cauterization, with the subsequent use of emollient enemata and opium suppositories. For the phagedænic chancroid, constitutional treatment is desirable, as in all other cases of phagedæna. Opium—one grain at night and one grain in the morning—is, I think, more beneficial than any other single remedy. In some cases it may be of advantage to remove the surface of a phagedænic or serpiginous chancroid by scraping with a scoop and then using as a caustic, bromine, permanganate of potassium, or caustic potassa; but I think that the hot iron is the best local remedy in these cases. Syphilization has been used for chancroid, but it is of no value.

In regard to the principal complication of chancroid, the bubo, it may be of two kinds, the simple or inflammatory bubo, which is nothing but an adenitis, or the true chancroidal or virulent bubo. I believe it to be impossible, when a bubo first makes its appearance, for the surgeon to say of which variety it is. Of late years I have seen many more examples of the simple than of the virulent bubo. Whether or not this is because the disease, like syphilis, is gradually becoming a milder affection than it was formerly, I cannot say.

In regard to the treatment of bubo, the surgeon should enforce rest in bed, if possible. Then counter-irritation should be employed very thoroughly. The best way is that suggested by Mr. Furneaux Jordan, of Birmingham, by applying the counter-irritant to the "next vascular area." The theory is that by irritating an adjacent part, the blood is caused to flow away from that originally affected. Counter-irritation is best effected by applying the tincture of iodine in the form of a broad horse-shoe around the inflamed gland, every day or every other day, so as to keep the part on the verge of vesication. The skin should, if possible not be broken, but if it is so, some soothing ointment must be applied, and the use of iodine suspended for a few days. Over the bubo itself, the dressing which I have found most satisfactory consists of equal parts of belladanna and mercurial ointments; it is a simple resolvent and anodyne application, and is agreeble to the patient. I have also used an ointment of iodoform over the part, but do not think it as efficient as the belladonna and mercury; nor do I think the application of blisters as satisfactory as the use of iodine. Pressure is another remedy which may be properly employed when the bubo is not painful, but which is ill-adapted to the acute inflammatory stage. If it is to be employed, pressure may be effected by applying a shot-bag over the bubo while the patient is in bed; or by fastening a soft sponge over the part with a spica bandage applied with the thigh flexed on the trunk. If the bubo suppurates, of course it should be opened. Various plans have been suggested, but I do not think there is anything as efficient as a moderately free incision; and the direction in which this is made is a matter of considerable importance. I find that practitioners generally open buboes in the line of Poupart's ligament, but I think that an incision in the long axis of the patient's body is the best, supplemented, if necessary, by small transverse incisions on one or both sides. If the lips of the wound are kept apart, so as to allow the pus to flow out readily, the process of healing is much more rapid. Multiple punctures have been employed in opening buboes, and the introduction of a a seton has also been suggested; in case phagedæna attacks the bubo, the use of the continuous hot bath has been proposed. My experience is here, too, in favor of the use of opium, locally and internally, and, if cauterization is necessary, the application of the hot iron. I think that there is an advantage, as regards the bubo, in a thorough cauterization of the original chancroid at the beginning. Bumstead and Taylor recommend that cauterization should be employed if it can be done in the first ten days; but if it is desirable in the first ten days, it seems to me to be proper at any period. These gentlemen believe that by early cauterization the patient will escape virulent bubo, and that even if an inflammatory bubo exists, its course will be favorably modified. I am aware that a directly contrary opinion is held by some surgeons, who believe that the risk of bubo is increased by cauterization, but, as far as my own experience goes, it confirms the teaching of Bumstead and Taylor.

If the surgeon is satisfied that he is dealing with a chancroidal or virulent bubo, simple incision is not sufficient. Here suppuration occurs first in the periglandular areolar tissue, and it is of great advantage to enucleate the infiltrated glands before they become disintegrated and inoculate the surrounding tissues with chancroidal matter. If the case is not seen until the whole wound has become inoculated, then I would slit up all sinuses, remove the thinned, overhanging skin, and cauterize the whole surface with nitric acid, the patient being under the influence of ether.

The third plan of treatment, which is the fashionable treatment just now, is the use of simple dressings such as I have advised for the after-treatment, without employing caustics. There is no doubt that healing will, in most of the mild, superficial chancroids met with at the present day, ultimately take place without cauterization, but I think the cure will be more certain, more rapid, and more likely to be free from complication, if the chancroid be cauterized in the way that I have recommended.

TREATMENT OF SYPHILIS.

Syphilis is a constitutional affection and demands constitutional treatment. The principal remedies are mercury and iodide of potassium. These have been given for many years, and yet it has never been satisfactorily determined in what way they produce their effects. Probably it is the safest to say they act by eliminating the syphilitic poison and producing absorption of the gummatous and inflammatory deposits. No doubt, according to modern theories, they might be supposed to act by destroying syphilitic germs, but that suggestion opens questions in transcendental pathology into which this is not the time to enter.

For the convenience of considering the treatment of syphilis, we may divide its course into the primary, secondary and tertiary stages.

The lesions of the primary stage are the initial lesion (or chancre) and the bubo which accompanies it. Now, in regard to the treatment of primary syphilis, I believe that the surgeon will do well to put his patient under mercurial treatment, provided that he is sure of his diagnosis. This view is opposed, however, by some authorities, for whom I have great respect. My practice is to give mercury, and the best form in which it can be given, in the primary stage, is the green iodide of protiodide. I have been in the habit of prescribing this preparation in pills with opium alone, or made up with a confection of opium as an excipient; it has the advantage that it can be used a long while without causing salivation, and it is, moreover, efficient. I think that this is the safest mode of treating syphilis in the primary stage, but no patient should be placed on a mercurial course unless the surgeon is well satisfied that syphilis is actually present.

In regard to the local treatment of primary syphilis, the principal point is cleanliness; but local treatment is not of much value. Iodoform may be used as a dressing for the chancre, as it may for the ulcerative lesions met with in the later stages of syphilis. Cauterization is of no service. I do not believe that secondary symptoms were ever prevented by cauterizing a chancre.

There is another form of treatment which has some evidence in its favor, and that is the excision of the chancre.

Until within a few years the view of surgeons was that a chancre should not be excised except under special circumstances, as when occurring on an elongated prepuce, but within recent years the excision treatment has been revived, particularly in Germany, and in this country it has been advocated by Dr. White and others. To those who, like myself, take the view that syphilis is a constitutional disease from the beginning, and that the initial lesion, chancre, is but its first manifestation, of course the excision treatment seems somewhat unphilosophical. I have no personal experience in this form of treatment, but the weight of evidence, from what I have been able to read concerning it, seems to me to be against it. This, moreover, appears to be the prevailing view among the leading specialists in venereal affections in New York.

As regards the bubo of syphilis, no special treatment is required, though I have sometimes thought that I have derived advantage from the application of iodoform ointment.

In the treatment of the secondary stage of syphilis, of course mercury is the great remedy. Iodide of potassium is used by some surgeons in the primary stage, but for secondary syphilis all are agreed to use mercury. It should be introduced gradually, to prevent salivation on the one hand and intestinal irritation on the other. I think the best way in which it can be used is by inunction. I recommend the patient to rub ordinary mercurial ointment, or an ointment of the oleate of mercury, into the inner side of the thighs, using fifteen grains each morning and night, half a drachm altogether in the course of the day. If this seem too much, the remedy can be suspended for awhile, and then used in diminished doses. Another good plan is to apply the ointment to the soles of the feet, wearing woolen stockings; the place of application should be frequently changed, so as to avoid the occurrence of mercurial eczema. Before each application, too, the skin should be thoroughly washed and dried. In cases of infantile syphilis, Brodie's plan of putting the mercurial ointment on the belly-band is a good one.

If a patient objects to inunction, then mercury must be given by the mouth. The old-fashioned blue-pill is one of the most efficient preparations, if it is given cautiously, or the iodide may be used, or the bichloride, which, however, I think less useful than the others. Mercurial fumigation is a good method of treatment in certain obstinate forms of cutaneous syphilis, but is too troublesome for ordinary employment. Another mode of administering mercury is by hypodermic injections, usually of from $\frac{1}{12}$ to $\frac{3}{8}$ gr. of the corrosive chloride, though almost any preparation of mercury may be used hypodermically. I do not think that this plan presents enough advantages to counteract its disadvantages, and believe that it should be reserved for exceptional cases.

For mucous patches, constitutional treatment must, of course, be continued, and as a local remedy, the solution of acid nitrate of mercury may be applied, being then followed by some simple dressing, such as black-wash, and iodoform afterwards. Another plan, recommended by Conradi, is to use a strong solution of nitrate of silver, and then to apply metallic zinc. For syphilitic sore throat, gargles of chlorate of potassium may be employed, or cauterizations with the liq. hydrarg. pernitratis; or dilute hydrochloric acid may be applied with an atomizer. For syphilitic iritis, I have been favorably impressed with Carmichael's mode of treatment, which consists in the administration of oil of turpentine in large doses. I have often used this with great advantage, but have, on the other hand, sometimes found it to fail, and have had to come back to mercury. The oil of turpentine is given in large doses (*f*℥j) three times a day, in emulsion with gum and sugar, with a few drops of the tincture of opium to prevent strangury. The most important point in the treatment of iritis, however, is the local use of atropia. For alopecia, cantharidal washes may be recommended.

In the tertiary stage of syphilis, iodide of potassium is the chief remedy. Mercury is useful in the treatment of the dry eruptions and of interstitial orchitis, but not in the gummatous affections, where iodide of potassium is preferable. At the same time tonics must be given, as indeed in the secondary and primary stages also. An expectant plan of treatment has been suggested for syphilis, but it is not to be recommended, nor would I favor hygienic and tonic treatment by itself, though in connection with specific treatment it is of great value. A patient who lives a regular life, avoiding the use of tobacco and alcohol, and at the same time pursuing a proper course of treatment, has a better chance of recovery from syphilis than one who neglects hygienic measures.

In giving mercury for syphilis, there are two plans of proceeding: one in which small doses are given continuously for a long time, as particularly advised by Dr. Keyes, of New York; and the other, which seems to me more philosophical, in which the drug is given "*coup sur coup*," that is, in successive courses with intervening intervals. The doses should be moderate, and salivation should be avoided. The best way is to give mercury cautiously until the symptoms are relieved, or a few weeks longer, and then to suspend it altogether. Then if there are any fresh symptoms the administration may be renewed.

It has been proposed by Mr. Venning, as a test to determine when syphilis has been removed from the system, to examine the condition of the inguinal glands. If there is any induration remaining, the patient is still syphilitic.

Iodide of potassium may be used very freely in syphilis. Formerly, five-grain doses were ordinarily given, but from eight to ten grains is now considered a fair dose to begin with, and in some cases much larger quantities must be employed. I am convinced, however, that the drug is often given in excessive amounts in ordinary cases of syphilis. I do not recommend large doses unless the disease fails to respond to smaller ones, or unless the symptoms, as in some cases of cerebral syphilis, are immediately threatening to life. The iodide may be given simply in water, or with the compound syrup of sarsaparilla, or with fluid extract of gentian, viz.:

Pot. iod., gr. viii–x.
Ext. gent. fl. ♏ xv.

With enough water to make a teaspoonful. Iodoform has been given internally, and homœopathic practitioners have employed gold, but neither appears to have any special value. Sarsaparilla used to be looked upon as an important remedy for syphilis, but I have never found that it was of any use whatever. A remedy strongly recommended by the late Dr. Sims was stillingia. Dr. Taylor speaks favorably of the erythroxylon coca. Hot baths are undoubtedly of use sometimes in syphilis. For hereditary syphilis, mercury and iodide of potassium, in doses suited to the age of the patient, and combined with tonics, and especially iron, are of use. If a syphilitic woman is pregnant, she should undergo a mercurial course, in hope of preventing infection of the fœtus.—*John Ashhurst, Jr., M. D., in Maryland Medical Journal.*

